# Crystal scatter effects in a large-area dual-panel Positron Emission Mammography system

**DOI:** 10.1371/journal.pone.0297829

**Published:** 2024-03-01

**Authors:** Rahal Saaidi, Mercedes Rodríguez-Villafuerte, Héctor Alva-Sánchez, Arnulfo Martínez-Dávalos

**Affiliations:** Instituto de Física, Universidad Nacional Autónoma de México, Ciudad Universitaria, Coyoacán, Mexico City, Mexico; Tsinghua University, CHINA

## Abstract

Positron Emission Mammography (PEM) is a valuable molecular imaging technique for breast studies using pharmaceuticals labeled with positron emitters and dual-panel detectors. PEM scanners normally use large scintillation crystals coupled to sensitive photodetectors. Multiple interactions of the 511 keV annihilation photons in the crystals can result in event mispositioning leading to a negative impact in radiopharmaceutical uptake quantification. In this work, we report the study of crystal scatter effects of a large-area dual-panel PEM system designed with either monolithic or pixelated lutetium yttrium orthosilicate (LYSO) crystals using the Monte Carlo simulation platform GATE. The results show that only a relatively small fraction of coincidences (~20%) arise from events where both coincidence photons undergo single interactions (mostly through photoelectric absorption) in the crystals. Most of the coincidences are events where at least one of the annihilation photons undergoes a chain of Compton scatterings: approximately 79% end up in photoelectric absorption while the rest (<1%) escape the detector. Mean positioning errors, calculated as the distance between first hit and energy weighted (assigned) positions of interaction, were 1.70 mm and 1.92 mm for the monolithic and pixelated crystals, respectively. Reconstructed spatial resolution quantification with a miniDerenzo phantom and a list mode iterative reconstruction algorithm shows that, for both crystal types, 2 mm diameter hot rods were resolved, indicating a relatively small effect in spatial resolution. A drastic reduction in peak-to-valley ratios for the same hot-rod diameters was observed, up to a factor of 14 for the monolithic crystals and 7.5 for the pixelated ones.

## Introduction

Breast cancer is the main cause of cancer mortality among women. Recent statistics have shown that the age-standardized incidence has reached levels as high as 112 per 100,000 population [1]. The detection of this malignant neoplastic disease at an early stage can improve the survival rate of breast cancer patients [2, 3]. Consequently, early detection of the disease is recognized worldwide as a priority in health care for reducing breast cancer mortality. Positron Emission Mammography (PEM) is a valuable medical imaging technique to diagnose breast cancer, monitor patients in follow-up studies, image therapeutic response and discover recurrence [4–6]. It is based on the determination of the 3D distribution of a radiopharmaceutical in target organs or tissues, in the same manner as Positron Emission Tomography (PET) [2, 3]. Successful early breast cancer diagnosis requires imaging systems with high sensitivity and high spatial resolution. A scanner with high sensitivity allows to reduce both the injected dose and scan duration while, at the same time, increasing the signal-to-noise ratio. Scanner sensitivity mainly depends on the solid angle coverage (the scanner’s detector arrangement) and photon detection efficiency (detector type and quantum efficiency). In a PEM system, sensitivity is partly achieved by using a pair of parallel plate detectors in close contact with the organ, slightly compressing the breast. A scanner with high spatial resolution is highly desirable since it would allow the identification of small-sized lesions [2]. Spatial resolution, however, is affected by several limiting factors that include positron range, geometry of the detection system (crystal dimensions, scanner architecture, etc.) and image reconstruction algorithms [7].

PET scanners commonly use indirect detection of the annihilation photons by coupling scintillation crystals to sensitive photodetectors [8]. Pixelated crystals are widely used for assembling detector blocks for PET scanners, attaining reconstructed spatial resolutions between 3 and 5 mm. However, this type of crystals presents several drawbacks, such as loss of sensitivity due to dead space between pixels, lack of information on depth of interaction (unless specialized detector configurations and readout electronics are used) as well as high costs involved in their manufacturing. In the last years, monolithic scintillator crystals have been successfully used with promising characteristics in terms of cost, sensitivity, spatial, timing and energy resolutions, that can overcome those of pixelated crystals [9]. However, in both detector configurations intra-detector Compton scattering can degrade the spatial resolution of the reconstructed images, introducing additional image blurring [10–15].

Photon interactions in the detector produce multiple locations where energy is deposited, leading to uncertainties as to where the first photon interaction occurred. When the crystal is pixelated, partial energy depositions may occur in multiple crystal elements, leading to incorrect positioning of the lines of response [13, 16]. A similar mechanism happens in a monolithic crystal, but in this case the 3D light distribution covers a larger area in the photosensor for a single photon track. In general, the extent of crystal scatter effects depends on the geometry of the detector [17].

Compton scattering is the dominant interaction mechanism for 511 keV photons in common inorganic scintillation crystals. For example, about 67% of the interactions of annihilation photons occur via this mechanism in LYSO. Its effect on image quality depends on the scintillation material and system geometry (mostly crystal size and thickness), scintillator mass density, detector separation that register the coincidence events and photon incident angle [10, 18].

Four different combinations of photon interactions are of relevance in PET (and thus, in PEM), classified in terms of the number of interactions and the energy deposited in the scintillator:

▪ Single photoelectric (PE) interaction, in which the photon is absorbed with 511 keV energy deposition.
▪Single Compton (CS) scattering, where an incident photon scatters only once in the crystal and it escapes the detector, depositing less than 511 keV.
▪Multiple Compton scattering occurring in different crystal positions, with the last scattered photon escaping the detector. In this case, the total energy deposited in the crystal is less than 511 keV.
▪Single or multiple Compton scattering ending in a photoelectric interaction, depositing the full 511 keV photon energy.

Only the first two combinations produce unequivocally correct first positions of interactions in the crystal, leading to unique lines of response (LORs).

Monte Carlo simulations are useful and effective tools for studying in detail the different types and combinations of photon interactions in detector materials. The free open-source software Geant4 Application for Tomographic Emission (GATE) is frequently used for simulating tomographic experiments for PET and Single Photon Emission Computed Tomography (SPECT) systems owing to its flexibility and well validated applications [19, 20]. Several works have carried out investigations on crystal scatter for PET scanners with different assemblies, ranging from simple geometries, like two opposing detectors, to more sophisticated multi-ring tomographic systems [10–14]. For example, Zhang et al. [10] found in a Monte Carlo simulation study using GATE that single interactions of 511 keV photons from a point source in two opposing 50×50×20 mm^3^ monolithic LYSO crystals, 10 cm apart, accounts to only 16.1%, but multiple Compton interactions are still useful in providing spatial information, provided that a low energy threshold of 350 keV is used.

In this work, we report the study of crystal scatter effects of a large area PEM scanner currently being developed in our laboratory, with different crystal configurations. To the best of our knowledge there is no detailed analysis of scattering effects in a large area PEM system in the literature. Also, the comparison between two crystal configurations (monolithic and pixelated) has not been done previously for such a system. The study was carried out with the GATE v8.2 code, which can provide information to identify the mispositioning events for the two crystal configurations. We present a comparison on the effect that Compton scattered events has on both monolithic and pixelated crystal arrays, not only using a uniform source distribution (simulating the breast), but with the simulation of an image quality phantom. The latter considered the geometry of a miniDerenzo phantom to quantify the effect of crystal scatter on reconstructed image quality, in terms of spatial resolution and peak-to-valley ratios (PVR).

## Materials and methods

### PEM system

The dual-head PEM scanner described as follows correspond to a real system being constructed in our laboratory. It is composed of two large area panels (17.6×17.6 cm^2^), each one assembled with 3×3 detector blocks of either monolithic or pixelated LYSO crystals. The monolithic crystal is a continuous LYSO scintillator of 57.4×57.4×10 mm^3^, while the pixelated one is a square array of 40×40 LYSO individual crystals (each of 1.35×1.35×10 mm^3^) separated by an ~90 μm specular reflector material VM2000 (Proteus Inc., Chagrin Falls, OH, USA) covering all sides of the crystal elements except the one facing the photosensor and crystal pitch of 1.44 mm. Both crystal types cover the active area of the photosensor. Each detector block is an assembly of the LYSO crystal (monolithic or pixelated) coupled by a 6 mm light guide to a position sensitive silicon photomultiplier (SiPM) array of 8×8 elements (ArrayC-60035-64P, SensL Technologies Ltd., now ON Semiconductor), with total area of 57.4×57.4 mm^2^. For this model, the active area of the individual SiPMs is 6×6 mm^2^, 7.2 mm pitch.

Optical grease couples the scintillator and the 6 mm thick light guide, while a 1 mm thick optical silicone couples the light guide and the SiPM, thus requiring a total of 7 mm optical material thickness between the scintillator and the photodetector. The latter was decisive to reduce artifacts when considering the pixelated crystals coupled to position sensitive SiPM, as reported by our group from experimental measurements in [21]. The light guide consists of a single 57.4×57.4×6 mm^3^ polymethylmethacrylate (Eljen Technology, Broadway, Sweetwater, TX) with a refractive index of 1.502 at 436 nm. The 1 mm thick silicone rubber (EJ-560, also by Eljen Technology), is a fully-cured polymer with a refractive index of 1.43 (wavelength not specified by the manufacturer).

It is interesting noticing that, although in both crystal types the scintillation light is emitted isotropically, its propagation occurs in a different manner. In the monolithic crystal, light spreads throughout the whole crystal volume, while in the pixelated one light is restricted to travel mainly inside the crystal pixel with reduced light sharing in the photosensor area [22].

**Fig 1:**
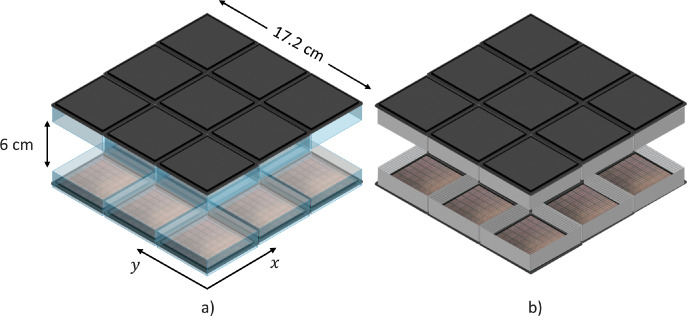
GATE geometry model of the dual-panel PEM with monolithic (a) and pixelated (b) crystals. The upper reflective layer in the crystals in b) was assumed transparent in this drawing for ease of visualization. Tomographic images reconstructed parallel to the (*x*, *y*) plane are referred to as in-plane.

The dual-plate system was simulated in GATE using the Cylindrical PET scanner model, which is a requirement for the tomographic image reconstruction program used in this work. The same detector response and geometry was assumed in the simulation as described by the real detector. The scanner geometry is illustrated in Fig 1, and its main characteristics are summarized in Table 1. All the simulations assumed a 6 cm panel separation, which corresponds to a medium level of breast compression [23].

**Table 1:** Characteristics of the dual-panel PEM scanner simulated in this work.

	Monolithic	Pixelated
Crystals		
Dimensions (mm^3^)	57.4×57.4×10	1.35×1.35×10
Elements in array	1×1	40×40
Pitch (mm)	-	1.44
No. per detector block	1	1600
Total number	18	28,800
Detector blocks per panel	9	9

In addition to the parameters used for the design of the scanner’s geometry, other parameters such as the physics processes and the digitizer detection chain were also considered as summarized in Table 2. Deadtime, coincidence timing window and energy resolution values correspond to experimental measurements carried out in the laboratory for our detectors. An energy window of [350, 750] keV was used in the simulation; the 350 keV lower threshold is the value used in clinical settings for the commercial dual-panel scanner Naviscan PEM Flex Solo II [24] (previously used also by our group for PEM experimental studies [25]) and for the MAMMI dedicated breast PET [26].

**Table 2:** Physics processes and digitizer chain used in GATE for the PEM simulation.

Parameter	Value
Physics (standard model)	Photoelectric
	Compton scattering (single or multiple)
Energy resolution at 511 keV	13%
Dead time (μs)	6
Coincidence timing window (ns)	6
Energy window (keV)	350–750

In all the simulations, hit and coincidence events were registered in ROOT files [27], followed by further off-line analysis with programs written in C++ and Matlab R2020a (The MathWorks Inc., Natick, Massachusetts, USA).

### Phantom geometry and sources

To isolate crystal scatter effects from any other contribution, the following considerations in the simulation were made:

▪ The phantoms were assumed air-filled to avoid photon attenuation and scatter in other bodies but the LYSO crystals.
▪ Non-collinearity and positron range effects were suppressed by considering sources of back-to-back emission of two 511 keV annihilation photons.
▪ Phantom activity was kept at minimum levels to avoid coincidence random events. An activity concentration of 3.7 kBq/ml was assumed, which has been reported as producing a linear count rate behavior in a commercial PEM scanner and is within the estimated patient background concentration (0.35 to 7.61 kBq/ml) reported in clinical studies [28, 29].

Two different phantoms placed at the center of the PEM’s field of view (FOV) were simulated:

- A cuboid shaped phantom of 15×10×6 cm^3^ containing a uniform source distribution. A total number of 3.7×10^6^ back-to-back emissions was considered.
- A miniDerenzo phantom with hot rods perpendicular to the plate detectors. A total number of 13.6×10^6^ back-to-back emissions was simulated.

### Interaction classification: Hits and coincidences

**Fig 2:**
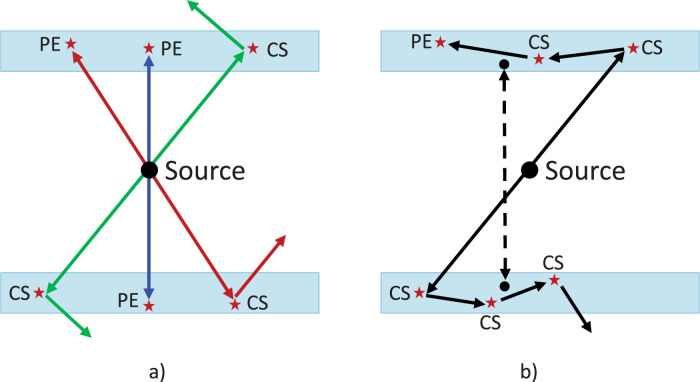
Examples of different combinations of coincidence events undergoing a) single interactions in the crystal leading to correct determination of LORs, b) multiple photon interactions in both crystals producing a mispositioned LOR (dashed line).

To investigate the probability distribution of coincidence detection events, the hits and coincidences branches of the ROOT files were analyzed. Fig 2A show cases in which both photons undergo a single interaction (SI) -either photoelectric or single Compton scattering, producing the correct LORs-, while Fig 2B exemplify multiple interactions (MI) in both crystals that typically require an energy weighted position calculation producing a mispositioned LOR (shown as a dashed line). Using this notation, the coincidences were classified in the data files.

The output parameters in the hits ROOT file, which register every photon interaction in the detector, enables correlating the full track of a coincidence event with a set of hits within the crystal. The final position assigned to a coincidence event is calculated in GATE as the energy weighted (EW) centroid of the hit positions, here referred to as EWP. For a given photon history, the common variable that allows relating hits and coincidences is the event identifier (eventID). For every registered coincidence, the EWP and deposited energy were known and, using the eventID, its interaction chain (that is, the particle track containing a full hits history) was recreated, including the first hit position (FHP) as well as the number and type of interactions in the LYSO crystal.

From the interaction chain obtained for every annihilation photon, the events were classified as a function of the number and type of interactions, that is, as single photoelectric absorption (PE), single or multiple Compton scattering (CS_1_, CS_2_ and CS_3_ for 1, 2 or 3 Compton scatterings), ending or not in photoelectric absorption.

### Positioning error in the crystal

For every registered coincidence event the Euclidian distance between the FHP and the EWP was calculated from their corresponding coordinates (*X*_*FH*_, *Y*_*FH*_) and (*X*_*EW*_, *Y*_*EW*_) retrieved from the hit positions of the track, as it serves as an estimation of the positioning error Δ*r*_*xy*_ of the corresponding LOR. These distances were calculated along the lateral direction (that is, *in-plane*), which is the direction that affects the most the correct determination of the LORs. From these data, histograms of positioning errors were calculated.

### Image resolution phantom

**Fig 3:**
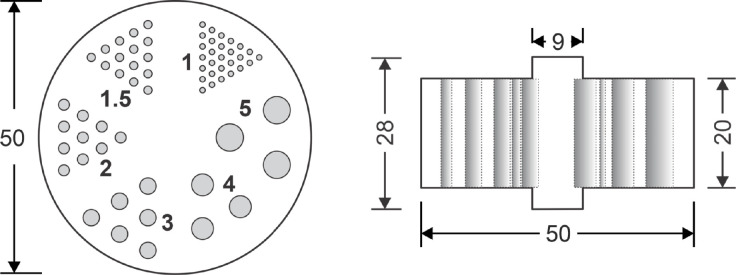
Transverse and axial views of the miniDerenzo phantom [25] filled with air. The phantom was placed in the middle of the PEM panels which were separated by 6 cm. All units are in mm.

To quantify the effect of crystal scatter in the image quality of reconstructed images, an air-filled miniDerenzo phantom of 50 mm diameter and 20 mm height was simulated containing hot cylinders of 1, 1.5, 2, 3, 4 and 5 mm diameter and arranged into 6 pie-segments [25]. For every pie segment, the center-to-center distance between adjacent cylinders was considered as twice the cylinder diameter (see Fig 3). As mentioned previously, back-to-back emission of two 511-keV gamma rays with activity concentration of 3 kBq/ml in the hot rods were considered.

A common qualitative assessment of spatial resolution with a Derenzo-type phantom is based on the minimum size of the hot rods that are visually distinguishable in the reconstructed *in-plane* image. However, for a given pie segment, hot rods may have different contrasts and, therefore, this appreciation may be uncertain. To overcome this ambiguity, the calculation of PVR is a well-established quantitative metric used to assess spatial resolution [14, 30–33].

In this work, to quantify the effect of crystal scatter on reconstructed images of the miniDerenzo phantom, the following parameters were calculated for the minimum size of all nearest hot rods of the same diameter that were visually distinguishable.

- PVR (average, minimum, and maximum values) along horizontal intensity profiles.
- Full-width-at-half-maximum (FWHM) in the *x* and *y* directions of asymmetric two-dimensional (2D) Gaussian functions fitted to every hot rod.
- Resolvability indices (*RI*) [21] to quantify the capability of the system to differentiate between independent hot rods.

The *RI* in the *x* and *y* directions were calculated as:

RI=FWHM/d (1)

where FWHM comes from the 2D Gaussian fit to individual hot rods and *d* is the distance separating their centers. When *RI*<1 the hot rods are distinguishable as separate entities; smaller *RI* values imply a better ability of the system to resolve the hot rods [34].

### Tomographic image reconstruction

A dual-panel PEM is known as a limited-angle tomographic system due to the lack of solid angle coverage for all the lines of response. Because of this lack of complete angle information, filtered backprojection cannot be performed [32, 35, 36]. In this work, tomographic image reconstruction was carried out with the public domain platform CASToR (Customizable and Advanced Software for Tomographic Reconstruction) using a List-Mode Ordered Subset (LM-OS) Maximum Likelihood Expectation Maximization (ML-EM) iterative algorithm [37]. The geometry macro files from GATE, together with the coincidences ROOT tree output, are used as CASToR’s input data through a conversion process. For a detailed description of the methodology see the S1 Appendix.

Normalization correction was applied during image reconstruction. Planar sources have been widely used to obtain normalization correction factors for ring scanners [31], for a pair of block detectors [38] and for dual panel PET systems [32, 39, 40]. In this work, we followed the normalization procedure described in [32] by simulating a planar source of back-to-back 511 keV photons, placed at the middle and parallel to the two detector panels covering the PEM’s FOV. The normalization image was calculated in an array of 174×174×24 voxels (each of 1×1×2.5 mm^3^).

The LM-OS ML-EM image reconstruction was carried out with all LORs (regardless of their angular distribution), using 2 iterations, 28 subsets, 174×174×24 voxels each of 1×1×2.5 mm^3^ (24 slices), with and without the application of an isotropic 1.5 mm FWHM 3D Gaussian post-reconstruction filter (GF).

## Results

As mentioned in the previous section, the activities in the simulated phantom studies were kept at minimum levels to avoid coincidence random events. In all cases, randoms accounted for less than 0.4% of the total registered coincidences.

### Photoelectric and Compton scattering contributions

For the cuboid phantom, the number of coincidence events registered for the monolithic and pixelated crystals were 3.29×10^5^ and 2.75×10^5^, respectively, which accounted for 1.18×10^6^ and 9.68×10^5^ hits. These results show a large difference in sensitivity: the monolithic crystal registered around 20% more interactions than the pixelated crystal. This can be explained by the extent of the monolithic crystal volume, ~1.12 larger than the volume of the pixelated crystal array.

For single events, approximately 45% of all the interactions in the LYSO crystal occur through a single photoelectric absorption. Single Compton scatter contribution is insignificant (<0.11%) as even the largest scattering angle has a negligible probability of depositing sufficient energy in the LYSO crystal to be accepted within the energy window. The rest of the interactions arise through Compton scatterings with a large probability of the series of events ending in photoelectric effect. For both crystal types, Compton scattering in the crystal ending in photoelectric absorption accounts for ~55% of the single events and, from this, the fractions corresponding to 1, 2, 3 and >4 scatterings were 0.449, 0.371, 0.038 and 0.142, respectively. Thus, in what follows, multiple interaction refers to a photon undergoing Compton scattering (CS_1_, CS_2_, CS_3_, CS_>4_, for 1, 2, 3, >4 scatterings) and ending in PE. The coincidence classification as SI or MI is based on the number of hits that the photons undergo in the crystal. For example, if a Compton scatter is followed by photoelectric absorption and both occur in the same element in a pixelated crystal, the interaction is treated as MI.

The coincidence events were classified according to the following combinations (with the percentage of these events listed in Table 3):

SI–SI: Both annihilation photons undergo SI

MI–SI: One annihilation photon undergoes MI and the other a SI

MI–MI: Both annihilation photons undergo MI

**Table 3:** Percent contributions of the different coincidence events classified as SI and MI in the monolithic and pixelated crystals.

Crystal	SI–SI	MI—SI	MI—MI
Monolithic	19.5%	49.4%	31.1%
Pixelated	20.7%	49.7%	29.6%

**Fig 4:**
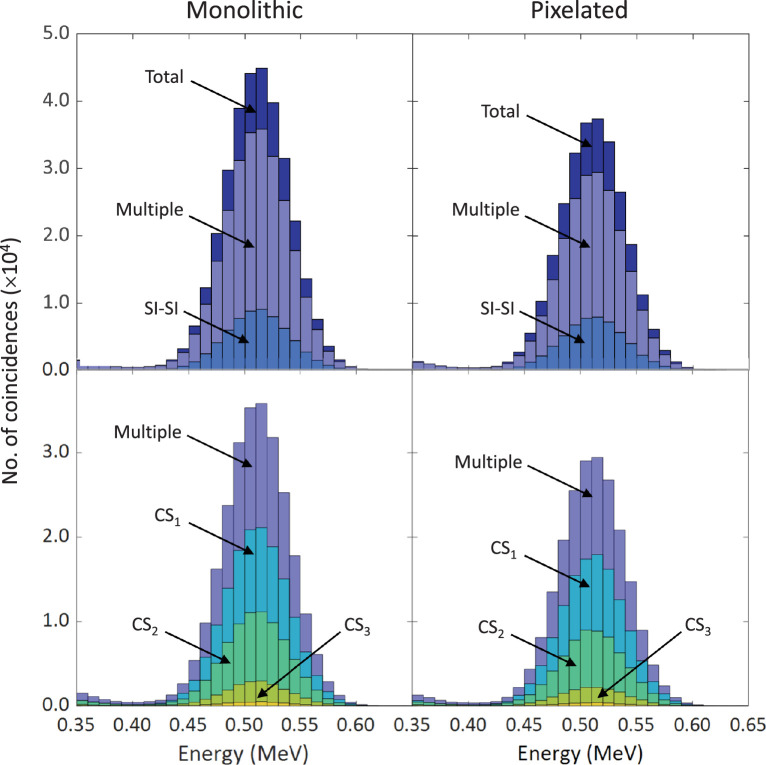
Energy spectra of coincidence events registered in both crystal configurations. Around 80% of the events are detected through multiple Compton scattering ending in photoelectric absorption. The histograms in the bottom row show the break-down of events where at least one of the coincidence photons underwent 1, 2 or 3 Compton scatterings (CS_1_, CS_2_, CS_3_).

In both crystal configurations the largest contributions to the MI–SI coincidences arise from CS_1_ and CS_2_ ending in PE, with fractions of 0.68 and 0.25, respectively. Fig 4 shows the energy distributions of the coincidence events classified as SI-SI or multiple interactions. Multiple, in this case, refers to all those events (MI-SI and MI-MI) where at least one annihilation photon undergoes a given number of Compton scatterings in LYSO. The well-defined shape of the photopeak in the spectra is due to the absence of photon interactions in the air phantom, and the negligible probability of multiple Compton scattering with the last scattered photon escaping from the crystal.

### Positioning error histograms

**Fig 5:**
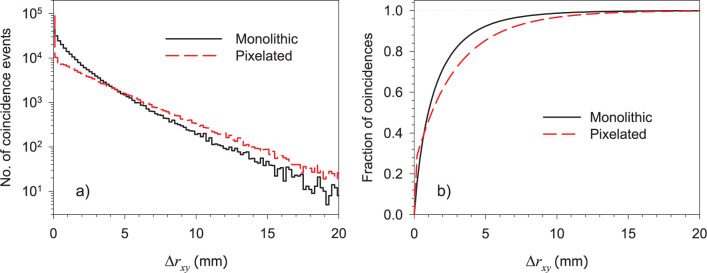
a) Positioning error (Δ*r*_*xy*_) histograms in semi-logarithmic scale and b) fraction of coincidence events positioned within Δ*r*_*xy*_ for both MI-SI and MI-MI in monolithic and pixelated crystals.

Fig 5A shows positioning error histograms as defined previously between the FHPs and the EWPs of interaction calculated in GATE for both crystal configurations and the cuboid phantom. The histograms were calculated for those coincidence events where at least one annihilation photon underwent multiple interactions (that is, events from Table 3 classified as MI-SI and MI-MI). For single interactions (SI-SI) the positioning error is equal to zero as the true first hit interaction position is the same as the energy weighted position.

The positioning error histograms in Fig 5A for monolithic and pixelated crystals present a drastic decrease at small (*x*, *y*) distances, with cusp-like shapes and long tails extending over 20 mm. The error distributions are different for the two detector types; the pixelated crystals show a narrower distribution with a longer extended tail, likely due to the crystal packing fraction; the dead gaps between crystal elements not only do not contribute to the detector response but have an impact in the photon tracks. The average of the positioning errors for coincidences that underwent multiple interactions obtained from the list mode data is 1.70 mm and 1.92 mm for the monolithic and pixelated crystals, respectively.

Fig 5B shows the fraction of events positioned with a distance *≤* Δ*r*_*xy*_ from the true first hit interaction position. For instance, 80% of the events are positioned within distances of 2.8 and 4.1 mm for the monolithic and pixelated crystals, respectively.

**Fig 6:**
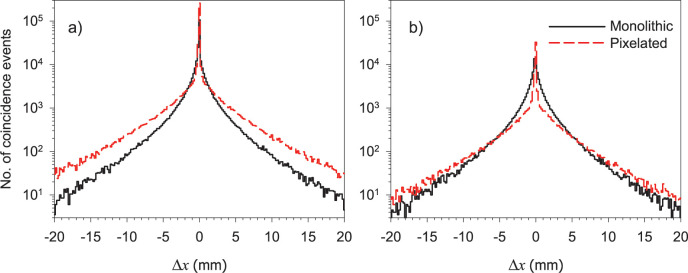
Δ*x* residual histograms in semi-logarithmic scale in monolithic and pixelated crystals considering a) both MI-SI and MI-MI coincidences, and b) only MI-MI coincidences.

Additional insights about the positioning errors can be obtained from an analysis of the residuals Δ*x* = *X*_*EW*_−*X*_*FH*_ (or Δ*y* = *Y*_*EW*_−*Y*_*FH*_) shown in Fig 6. Since multiple interactions occur randomly, it is expected that the distributions of the residuals in any direction would be the same. For this reason, in Fig 6A we only show the histograms of the residuals along the *x* direction for all multiple interaction coincidences (MI-SI and MI-MI). Furthermore, to emphasize the contribution of Compton scattering events, Fig 6B shows histograms of the residuals for the MI-MI coincidences only. The histograms also have cusp-like shapes and long tails extending over 20 mm, with different behavior depending on crystal configuration.

### Crystal scatter effects in image quality

**Fig 7:**
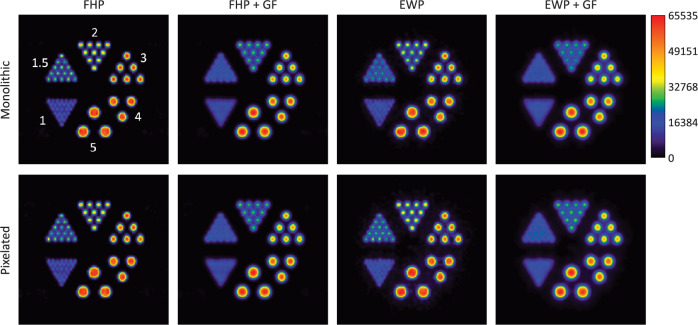
LM-OS ML-EM reconstructed images of the miniDerenzo phantom considering first hit (FH) and energy weighted positions (EW), with and without a post-reconstruction Gaussian filter (GF).

**Fig 8:**
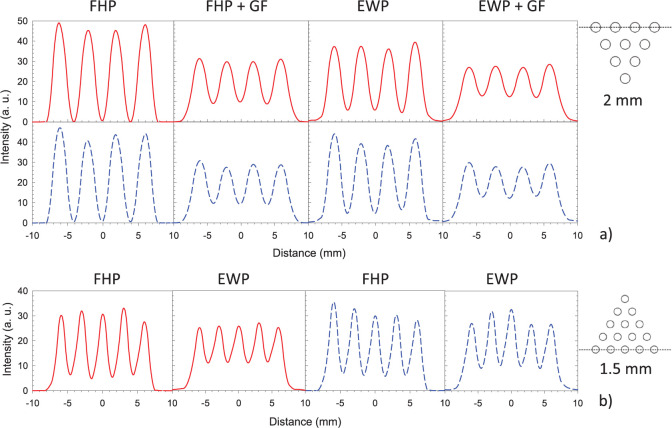
Horizontal intensity profiles across hot rod diameters of 2 mm (a) and 1.5 mm (b) from the reconstructed images shown in Fig 7 for the monolithic (red continuous lines) and pixelated (blue dashed lines) crystals. For ease of comparison, the profiles share the same maximum intensity for each rod diameter.

Fig 7 shows reconstructed images of the miniDerenzo phantom (average of slices 11 to 14) using the first hit and energy weighted positions for both crystal types. It can be observed that in all cases the 2 mm diameter hot rods can be resolved. This is not the case for the 1.5 mm diameter hot rods since they can be resolved only when using FHP without gaussian filter and is also confirmed through an inspection of horizontal intensity profiles extracted across hot rods of 2 and 1.5 mm diameter that where were visually resolved (shown in Fig 8).

A quantitative analysis through the calculation of the FWHM in the *x* and *y* directions, PVR and RI for the smallest hot rods that can be visualized, is shown in Table 4.

**Table 4:** Analysis of the reconstructed images (1.5 and 2.0 mm diameter hot rod pies) using either first hit or energy weighted positions. The errors correspond to ±1σ.

Rod ∅ (mm)		FWHM (mm)		RI		PVR	
		x	y	x	y	Average (min, max)	
2.0	Monolithic						
	FHP	2.02±0.11	2.05±0.23	0.507±0.028	0.513±0.058	78.5±51.7	(26.3, 165.3)
	EWP	2.14±0.12	2.35±0.19	0.536±0.030	0.587±0.048	5.6±1.4	(4.1, 7.8)
	FHP^†^	2.37±0.11	2.51±0.12	0.593±0.028	0.629±0.030	3.1±0.2	(2.9, 3.4)
	EWP^†^	2.56±0.29	2.83±0.31	0.641±0.073	0.708±0.078	2.0±0.1	(1.8, 2.2)
	Pixelated						
	FHP	2.03±0.10	2.10±0.22	0.506±0.025	0.525±0.055	42.7±14.5	(18.1, 58.4)
	EWP	2.11±0.14	2.29±0.24	0.528±0.035	0.571±0.060	5.7±2.6	(1.05, 7.98)
	FHP^†^	2.35±0.16	2.54±0.23	0.588±0.040	0.634±0.058	3.0±0.2	(2.67, 3.22)
	EWP^†^	2.49±0.31	2.74±0.29	0.623±0.078	0.684±0.073	2.1±0.2	(1.80, 2.30)
1.5	Monolithic						
	FHP	1.47±0.14	1.71±0.29	0.493±0.035	0.571±0.073	3.1±1.3	(1.2, 5.0)
	EWP	1.80±0.39	2.08±0.57	0.600±0.098	0.694±0.143	1.7±0.6	(1.0, 2.4)
	Pixelated						
	FHP	1.46±0.16	1.69±0.26	0.488±0.040	0.565±0.065	2.6±0.9	(1.3, 4.1)
	EWP	1.65±0.27	1.89±0.37	0.550±0.068	0.633±0.093	1.9±0.6	(1.3, 2.7)

^†^ With 1.5 mm FWHM isotropic Gaussian post-reconstruction filter.

### Spatial resolution

For the 2 mm diameter hot rod pie, the goodness of the 2D Gaussian fit (quantified through the Coefficient of Determination, *R*^2^, metric) of each hot rod image was >0.98. The FWHM values in the *x* and *y* directions are consistently different, being larger by approximately 10% in the *y*–direction. This might be due to the limited-angle coverage imposed by the PEM geometry and data acquisition. For the assumptions considered in this work, no significant difference can be observed visually between the two crystal types; both provide the same performance.

The smallest values of the resolvability indices correspond to images produced with FHP-no Gaussian filter; in all cases the indices are smaller than those produced with energy weighted positions.

### Peak-to-valley ratios

For the 2 mm diameter rods, the FHP produces the best PVR, with a larger value for the monolithic crystal. When no post-reconstruction Gaussian filter is applied, there is a drastic reduction in PVR by more than a factor of 14. The same is observed for the pixelated crystal, although the change is only by a factor of 7.5. When the post-reconstruction Gaussian filter is applied, the FHP images still show an important improvement in PVR (by approximately 50%), although the difference is less notorious.

The same analysis was performed with the 1.5 mm diameter hot rod pie, where the rods were visually distinguishable (summarized also in Table 4). Only two cases for each detector were selected where no post-reconstruction Gaussian filter was considered. The 2D Gaussian fits were not as good as for the 2 mm diameter hot rods, varying from 0.888 up to 0.975. Nevertheless, in this case the same conclusions were reached as with the 2 mm diameter hot rods: the most important effect of crystal scatter leads to a ~50% PVR reduction, and resolvability indices were smaller (thus, improving the capability of the system to resolve small hot rods) for images reconstructed using first hit positions.

## Discussion

Crystal scatter effects in PET detectors have been studied and reported in the last years, particularly for systems built with pixelated crystal arrays [10–12, 14, 16, 30, 41–43]. Lately, the use of monolithic crystals has attracted the attention of the scientific community due to their potential to perform in a similar manner (and, in some cases, even better) to pixelated crystal-based detectors. Therefore, it is important to characterize their performance. In this work we approached the study of crystal scatter in a PEM scanner by suppressing the influence of other physical factors like attenuation and scatter in the phantom material, positron range and non-colinearity of annihilation photons for both crystal structures. This allowed a direct comparison of crystal scatter effects on reconstructed image quality when using either monolithic or pixelated crystals in a large area dual-panel system.

Zhang and collaborators [10] reported Monte Carlo simulation studies of inter-crystal scatter effects on sensitivity and spatial resolution for two opposite LYSO crystal arrays with a square cross section similar to the individual detector blocks assumed in this study, and twice the crystal thickness. The probability that at least one coincidence gamma ray undergoes intra-crystal scatter is slightly larger (85%) in Zhang and collaborators’ work compared with our calculations (80%), likely due to the different design of our PEM system, consisting of an array of thinner crystals (10 mm thick) covering a larger field of view. Their results indicate that coincidence events that undergo multiple interactions significantly increase the tail of the spatial resolution (quantified through the full-width-at-tenth-maximum of a point source) rather than the FWHM. This can be correlated with our calculation of the cumulative distribution of the positioning errors shown in Fig 5B. The shape of these distributions (a sharp rise followed by a long asymptotic trend) is an indication of large tails; large Δ*r*_*xy*_ values can be due to the large area covered by the PEM panels. From these distributions it can be concluded that crystal scatter effects are more important for detectors with pixelated crystal arrays. This result is also consistent with our observations of the miniDerenzo phantom reconstructed images, where PVR is significantly altered if using coincidence energy weighted positions, with a relatively small effect in the resolvability index that quantified the spatial resolution of our system. The spatial resolution results obtained with the miniDerenzo phantom simulations indicate that the FHP provides the best capability of the PEM to resolve the hot rods. In the more realistic scenario, when using the energy weighted position with Gaussian filter, the resolvability index was well below 1.

Likewise, Hsu and collaborators [11] reported inter-crystal experimental studies with an innovative dual-panel PET made up of 3D position-sensitive (PS) scintillation detector with 1 mm^3^ isotropic spatial resolution. The capability of their system to track single interaction events in their 3D-PS detectors permitted to fully classify PE-PE events and to correct for event mispositioning for coincidence events that underwent single-scatter followed by PE absorption. Considering these coincidence events during image reconstruction, improvement in both sensitivity and contrast-to-noise ratio was found, in agreement with our observations when correcting for mispositioned events using first hit positions. Contrast deterioration in reconstructed images for other PET systems when using inter-crystal scatter coincidence events have also been reported for fully tomographic systems [12].

It is worth noticing the high quality of the reconstructed images produced by CASToR, in spite of using limited-angle data sets produced by the inherent geometry of the PEM system. In a real PEM study, a degradation in image quality would be expected due to the contributions of all the factors suppressed in the simulation to isolate crystal scatter effects. A collateral contribution of this work is the simplified method developed to associate virtual crystal arrays to monolithic crystals. As mentioned previously, CASToR operates on coincidence data produced with systems based on cylindrical PET geometries using pixelated crystal arrays. The conversion of (*x*, *y*) continuous positions of interaction in the monolithic crystal to a virtual crystal array based on the detector’s intrinsic spatial resolution is a straightforward procedure. Working in GATE’s local system of coordinates associated to each detector block facilitates the creation of virtual crystal identifiers (IDs). Rewriting the ROOT coincidence tree files considering these virtual crystal IDs is relatively easy and allows the user to test the conversion algorithm in a simplified manner using the same ROOT tools.

In all our calculations, DOI information was not considered since it does not play a very important role in the final reconstructed image of the PEM system due to the following main reasons: a) Our crystals are only 10 mm thick, relatively thin compared to other systems reported in the literature, b) In the iterative reconstruction algorithm for our system, we consider thick (2.5 mm) transverse slices, i.e., voxel size 1×1×2.5 mm^3^. By using geometrical considerations, the system matrix weights are much smaller for oblique LORs compared to less oblique LORs, and c) other research groups developing dual panel systems which do not incorporate DOI information report image resolutions on the order of (or even under) the crystal size [5]. Shi et al. [6] have found that not including DOI information in the reconstruction for a dual-head system like ours does not change the in-plane resolution (parallel to the detector), but it only affects the axial resolution (perpendicular to the detector). Our results agree with those publications.

This study intends to isolate crystal scatter effects from other physical factors affecting PEM image quality and quantification. It is difficult to anticipate the impact of positron range, photon non-collinearity, as well as attenuation and scatter in the breast. For instance, positron range depends on the energy distribution of positrons, which are different for each radionuclide. Energetic positrons, as those emitted by ^68^Ga, will have a larger impact in spatial resolution, as has been experimentally reported by our group in a previous work [44]. Photon non-collinearity will have a minor impact due to the proximity of the detector panels. Scatter and attenuation will depend on object thickness and material composition. Compton effect is the main interaction mechanism of 511 keV photons in water (~99.7%). However, the mean free path of photons in water is about 10.4 cm. In a typical 4.5 cm breast thickness, attenuation of 511 keV photons is approximately 35%.

An important consideration is that our results assume ideal positioning determination of photon interactions in the crystals, an assumption particularly important for the monolithic scintillators. The lack of scintillation photon production and transport in our simulations is likely responsible for the extremely narrow peak of the positioning error histograms distributions at very small positioning error values. For example, Gonzalez-Montoro et al. [45] have reported measured intrinsic spatial resolution of 0.7 mm FWHM for 48×48×10 mm^3^ monolithic LYSO crystal. This reported resolution includes the scintillator light distribution inside the crystal, which our study does not. Additionally, an important issue to consider in a real detector is the compression effect in the positioning towards the edges of the monolithic crystals and hot spots visible in their corners, as reported by Stockoff and collaborators [46]. These effects depend strongly on the positioning algorithm used with real experimental data, from the simplest center-of-gravity method up to more sophisticated iterative or artificial intelligence algorithms.

An overall comparison of the performance of monolithic and pixelated crystals is complicated because of the multitude of parameters involved in their design. In our case, where these design parameters are fixed (packing fraction, crystal size, etc.) monolithic crystals seem to have a slight advantage over the pixelated ones. Our analysis shows that both types of crystals have similar performance in terms of spatial resolution and resolvability index. Pixelated crystals have an advantage in terms of low distortion characteristics and slightly better PVR values, while monolithic crystals perform better in terms of sensibility and have lower positioning errors. However, monolithic crystals have a huge advantage in terms of price, with the added benefit of the possibility of retrieving DOI information, if so desired. These last factors become even more important if the final design involves covering a large FOV, as in our case. A good example of this is a very recent proposal to build a huge flat panel total-body PET scanner [47], where Monte Carlo simulation plays a significant role in the design decisions.

The varying degrees of how all the above-mentioned parameters contribute to the final image quality in PEM require separate studies on their own and are the subject of future work.

## Conclusions

In this work we report a detailed analysis of the effects of crystal scatter of the 511 keV annihilation photons and compare two crystal configurations (monolithic and pixelated) in a large-area dual-head PEM system for the first time. The probability that both coincidence photons suffer a single photon interaction in opposite detectors, leading to a correct positioning of the LORs, is 19.6% and 20.7% for the monolithic and pixelated crystals, respectively. There is a weak dependence of this probability on crystal configuration. The probability that at least one of the annihilation photons undergoes a scattering event in the crystal accounts for ~80%, in agreement with what has been reported by Zhang et al. [10] for a similar LYSO pixelated crystal. Positioning error histograms for monolithic and pixelated crystals showed cusp-like shapes with long tails and slightly different extensions, producing mean values of 1.70 mm and 1.92 mm, respectively. Reconstructed spatial resolution of a miniDerenzo phantom with a list-mode iterative reconstruction algorithm was not significantly affected by crystal scatter. An important reduction in PVR was observed; in the extreme case scenario, reduction by factors of up to 14.0 and 7.5 were calculated. This drastic reduction due to crystal scatter can preclude activity quantification of small lesions in PEM studies, unless corrections are applied to mispositioned scattered events. Our results point out that monolithic crystals have several benefits over pixelated crystals for the assembly of dual-panel PEM systems. Future work might include introducing all the effects involved in PEM imaging in a more realistic manner.

## Supporting information

S1 Appendix GATE to CASToR data conversion. Conversion process of GATE data for pixelated and monolithic crystals into CASToR’s input data.
(PDF)
