# Supplementary material for: Crystal scatter effects in a large-area dual-panel Positron Emission Mammography system
Source: PLoS One. 2024 Mar 1;19(3):e0297829. doi: 10.1371/journal.pone.0297829 (PMC10906883; doi:10.1371/journal.pone.0297829)
Supplement: S1 Appendix — Conversion process of GATE data for pixelated and monolithic crystals into CASToR’s input data. (PDF) [file pone.0297829.s001.pdf]

GATE to CASToR data conversion

CASToR can be used with input data from GATE simulations as long as: a) the scanner is defined using a Cylindrical PET geometry, and b) the detector blocks are assumed to be assembled with pixelated crystals [29]. To comply with the pixelated crystal requirement, while simulating monolithic crystals, in this work a special conversion procedure was implemented to transform continuous positions of interactions (x, y) to discrete crystal identifiers and used as CASToR's input files. The process was different for each crystal type.

Pixelated crystals

Since our investigation involved the retrieval of the first hit position for all the coincidence events, a C++ program was developed to convert the first hit coordinates (x_0, y_0) from the ROOT file of the pixelated crystal (in cm) to a discretized crystalID considering the original 40×40 LYSO crystal array in the PEM geometry. An output file was created in ROOT format and used as input for CASToR, together with the original GATE geometry file.

Monolithic crystals

This case required a special data handling to convert either the continuous (x_0, y_0) first hit or (x, y) energy weighted positions in the monolithic crystal to a virtual crystal array considering 1×1 mm^2 pixels. A 1 mm pixel size was selected to reflect the expected in-plane intrinsic spatial resolution of the PEM system. In this way, a virtual 58×58 LYSO crystal array was created for the monolithic scintillator and, for every coincidence event, the new crystalID together with all its attributes were rewritten in ROOT format. Additionally, a new PEM geometry file was developed assuming arrays of detector blocks made up of virtual 58×58 LYSO crystals (1 mm crystal pitch); these newly created files were used as CASToR's input data. Table S1 summarizes the procedure followed to create CASToR's modified input files for both, pixelated and monolithic crystals.

Table S1. Summary of the input files used for reconstruction with CASToR for both monolithic and pixelated crystals. Pitch, EWP and FHP refers to crystal pitch, energy weighted and first hit positions, respectively.

Crystal	Position	Cylindrical PET scanner geometry file	Coincidence ROOT tree file
Pixelated	EW	Original, 40×40 crystal arrays, 1.44 mm pitch	Original
Pixelated	FH	Original, 40×40 crystal arrays, 1.44 mm pitch	Modified with new crystalIDs calculated using FHP for every coincidence
Monolithic	EW and FH	Modified, 58×58 crystal arrays, 1 mm pitch	Modified with new crystalIDs calculated using EWP (or FHP) for every coincidence
